# Children’s Understanding of Topological Relations

**DOI:** 10.1162/opmi_a_00194

**Published:** 2025-03-03

**Authors:** Sami R. Yousif, Lily B. Goldstein, Elizabeth M. Brannon

**Affiliations:** Department of Psychology & Neuroscience, University of North Carolina at Chapel Hill, Chapel Hill, NC, USA; Department of Psychology, University of Pennsylvania, Philadelphia, PA, USA

**Keywords:** topology, geometry, spatial cognition

## Abstract

A core aim of developmental cognitive science is to uncover the basic building blocks of human thought. For instance, work revealing that even young children, adults without formal education, and distant animal species are sensitive to basic Euclidean properties indicates that humans may be endowed with some primitive understanding of Euclidean geometry. But what about other forms of geometry? Here, we explore children’s sensitivity to topological spatial forms. We show that children, like adults, spontaneously distinguish and match items in accordance with their topological relations. As well, we show that children’s judgments about object similarity are remarkably consistent with adults’, indicating stability in object concepts throughout the lifespan. Finally, we compare children’s sensitivity to various topological forms with their sensitivity to geometric properties like curvature, perpendicularity, and symmetry, and find that while there is some variability in performance across all the features tested, overall performance for geometric vs. topological is comparable. Collectively, these findings suggest that even young children have an intuitive understanding of topological relations and suggest that topological relations may be among the building blocks of human visuospatial representation.

## INTRODUCTION

Whether it be the carpentered walls of your home, the skyscrapers in the city outside, or the web of roads and transit systems connecting our towns and cities, you are at virtually every moment of your life surrounded by evidence of the extraordinary human ability to represent and manipulate space. But these achievements in architecture, engineering, and design have humble foundations. At the root of these capacities must exist some core building blocks of spatial representation. What are those building blocks—and how do they develop in the early years of life?

**Figure 1.**
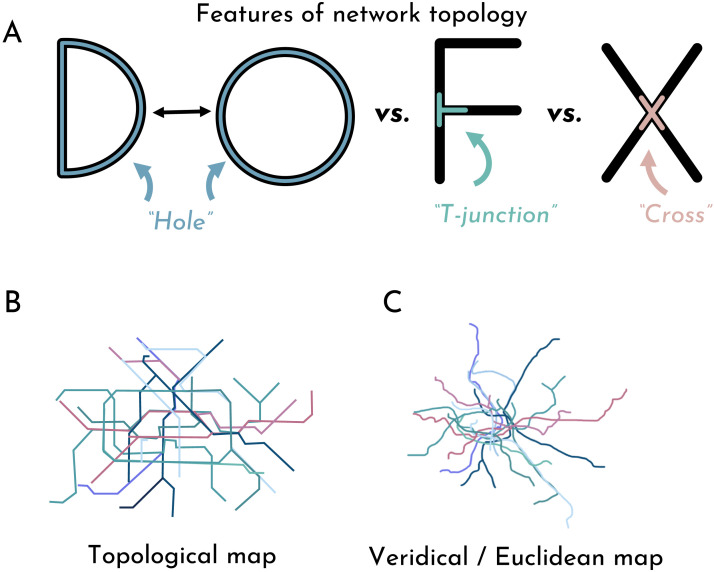
**(A)** A visual description of topological features of networks. **(B)** An example of a topological map, here of the city of Berlin. **(C)** An example of that same map stretched out to preserve metric detail.

In contrast with much work in developmental science which has emphasized the use of Euclidean geometric knowledge (Dehaene et al., 2006; Dillon et al., 2013; Lee et al., 2012; but see, e.g., Huey et al., 2023; Kenderla et al., 2023), here we explore sensitivity to a different form of geometry, *topology*. Specifically, we explore sensitivity to features of topological networks (see Figure 1A; see also Yousif & Brannon, 2024, 2025). Topology is the branch of mathematics concerned with spatial relations—physical properties that remain invariant under deformations such as stretching, twisting, and transforming objects. Whereas Euclidean geometry concerns itself with precise distances and angles, topological representations emphasize coarse structure. For instance, famously, a donut and a standard coffee mug are identical from a topological perspective, despite being quite different in their surface features. They are topologically the same in the sense that they both are bounded objects with only one hole.

The math of topology describes more than just bounded objects, however. Relations and networks can also be described topologically. Imagine, for example, simple mazes in the form of various English letters. A maze in the shape of the letter ‘D’ is functionally identical to a maze in the shape of the letter ‘O’: Both mazes are functionally just loops without any turns. Contrast that with the letters “F” and “X”. Each of these letters has a vertex where you would be forced to make a choice. Those choice points are functional. At the intersection of the “F” where the smaller horizontal line collides with the vertical line, there are always at least two different paths you could go down, no matter where you came from. This “three-point vertex” is sometimes called a “T-junction”. At the intersection of the “X”, there are always at least three different paths you could go down. This “four-point vertex” could be described as a “cross”. Importantly, three-point vertices and four-point vertices are both qualitatively different from two-point vertices (or “L-junctions”) in the sense that, while one might make a turn at a two-point vertex, one need not make a *decision* at a two-point vertex. This is what the topology captures: The functional parts of spatial structures which are relevant to how one would navigate the space, or how information would pass through the network.

Spatial representations which prioritize topological relations over properties like length and angle are common. Take the Berlin transit map, as seen in Figure 1B. This map is a topological map in the sense that it abstracts away from metric detail to instead place emphasis on the intersections between lines: It matters where two lines cross, but it does not matter how much distance there is between two crossings. The junctions, in other words, are the primary functional unit of these maps.

These days, such maps are common in virtually every major metropolitan area in the world—and for good reason. These maps are descriptive yet easily understood. In Figure 1C, the same map is depicted but with all of the metric detail preserved. While this latter, metric-detail-preserving map may be a more accurate representation of the space, the topological map is in many respects much easier to process. This simple fact that topological maps are intuitive, even without explicit understanding of topological representation, suggests that humans may have a natural proclivity for representing information in a topological form. It is through this lens that we explore children’s sensitivity to topological relations (i.e., the “junctions” which bind network spaces together).

### “Intuitive Network Topology”

Prior work has investigated sensitivity to object topology in adults (see, e.g., Chen, 1982, 2005; Zhou et al., 2010), primarily through the study of visual perception. This work has revealed not only that the visual system represents topological structures such as closure (Chen, 1982, 1990; but see Rubin & Kanwisher, 1985), but that topology influences other visual processes including apparent motion (Chen, 1985) and number perception (He et al., 2015) and also cognitive processes such as working memory (Wei et al., 2019). Related work has shown that children are also sensitive to differences in object topology (Chien et al., 2012; Kibbe & Leslie, 2016). In fact, sensitivity to object topology has been observed in species as distant as bees (Chen et al., 2003). These findings demonstrate that sensitivity to topology may be deeply ingrained, foundational to spatial representation throughout the human lifespan and perhaps even across the animal kingdom.

Our work focuses on a different kind of topological representation—not the topology of objects, but the topology of networks, or relations. In other words, we examine not the sort of topology which is concerned with mugs and donuts, but the sort of topology that is featured in most transit maps (see Figure 1B). Specifically, we are interested in whether children differentiate and match letter-like forms based on topological features like T-junctions and holes (see Yousif & Brannon, 2024). Recent work has demonstrated that, as with object topology, adults are broadly sensitive to network topology; they readily distinguish, match, and even remember network forms in accordance with their topological relations (Yousif & Brannon, 2024). This sensitivity is not limited to deliberate reasoning tasks, however. Differences in network topology influence rapid visual identification, visual search, and even number estimation (Yousif & Brannon, 2025).

Based on this evidence, we have argued that topological relations comprise a functional “language” of representing spatial relations that people intuitively “speak”. But how deeply ingrained is this language? Is it acquired through formal education, or, like sensitivity to Euclidean geometry (see Dehaene et al., 2006), does it naturally develop early in life?

### Topology as a Building Block of Spatial Representation

Even humans without formal education understand basic Euclidean geometric concepts like angle and distance as well as more complex geometric concepts like symmetry, perpendicularity, and centrality (Dehaene et al., 2006)—perhaps the most striking demonstration that there are indeed core building blocks underlying spatial knowledge. It may be unsurprising, then, that children are also sensitive to these Euclidean properties (though there is uncertainty about exactly which properties children are sensitive to; see Dillon et al., 2013; Lee et al., 2012; Yousif & Lourenco, 2017). Yet research on “core geometry” has largely focused on Euclidean geometry, at the expense of representational forms like topological relations (but see Dehaene et al., 2006; Gao & Hu, 2024; Huey et al., 2023). There is an opportunity, then, to understand the building blocks that support the understanding of topological spatial representations like transit maps (see Figure 1B).

Relatedly, there has been interest in the idea of a geometric “language of thought” (see Sablé-Meyer et al., 2021, 2022; see also Al Roumi et al., 2021; Amalric et al., 2017). If there is such a representational system, what are the core spatial concepts that comprise this language? One virtue of topological representations is that they can capture spatial structure with relatively few components: T-junctions, crosses, and holes are sufficient to describe the vast majority of path networks that we regularly encounter. Another virtue is that topological forms may play a role in spatial representation at the level of perceptual systems *and* at the level of navigational systems. As for perception: Yousif and Brannon (2025) showed that topological forms like T-junctions and holes are perceived rapidly and influence a range of visual processes from search to number estimation (thus raising the possibility topological relations are building blocks of visual representations, e.g., via “image grammars”; Lande, 2024). As for navigation: it has long been known that the errors people make when drawing out mental maps of unfamiliar environments are consistent with the use of topological representation (Byrne, 1979; Moar & Bower, 1983). Thus, if there is a geometric language of thought that spans perception and navigation, then topological relations seem likely to feature prominently in that language. If children, like adults, are sensitive to these basic relations, it may bolster the claim that topological relations are a core part of how we represent space (rather than being reliant on explicit, learned geometric knowledge, for instance).

### Current Study

Here, we examine (1) whether children are broadly sensitive to topological relations (per Yousif & Brannon, 2024; Experiment 1), (2) to what extent their judgments about topological relations resemble those of adults (Experiment 2), and (3) whether or to what extent network topology is related to other notions of topology, or other “core” geometric knowledge (Experiment 3). The overarching aim of this study is to ask whether young children appreciate network topology features to determine whether topological knowledge is a byproduct of education or a part of our foundational cognitive toolkit. If we find, for instance, that older children discriminate and match forms based on their topological structure, but younger children do not, this may indicate that knowledge of topological relations in some way depends on concepts that children acquire through formal education. In contrast, if we find that even young children possess some basic proclivity for discriminating and using topological forms, this may indicate that topological knowledge is early developing—which may prompt future work exploring just how early children exhibit such knowledge.

In a first experiment, we used an odd-one-out paradigm to test whether children discriminate based on topological relations. In a second experiment, we tested whether children match forms based on topology or surface features, and then we evaluated how well those judgments correspond with adult judgments for the same items. In a final experiment, we again used an odd-one-out paradigm to evaluate children’s performance discriminating based on topological features relative to their performance discriminating geometric features (borrowing some items from those used by Dehaene et al., 2006).

## EXPERIMENT 1: ODD-ONE-OUT TASK

First, we explored whether children are broadly sensitive to topological relations. Children completed an “odd-one-out” task modeled after the ‘intruder’ paradigm used by Dehaene et al. (2006) and more recently by Yousif and Brannon (2024). We created sets of stimuli that each contained one item that differed topologically from the rest. Children were shown these sets of stimuli, and on each trial were asked to identify which item was not like the others. Successful identification of the odd-one-out would indicate that children are sensitive to topological structure.

### Methods

#### Participants.

100 children (*M*_age_ = 5.96; *SD*_age_ = 1.18) completed the task; 25 of each age from four (four years, zero days) to seven (seven years, 364 days). Three additional participants were excluded because they did not complete the task (we pre-registered that we would exclude participant without a complete data set). We elected to recruit 100 children, reasoning that 25 children/group would be sufficient to detect any meaningful developmental change. Children were tested online (*n* = 62; recruited via a university developmental database) or in-person at a local museum (*n* = 38). Here, and for all subsequent experiments in this paper, we tested children as young as four years old because this was the youngest age capable of reliably completing these tasks in this form (i.e., in a way that would allow for direct comparison with existing adult data). We tested children as old as seven years old so that we could see whether or to what extent sensitivity to topology changes as children begin their formal education. The sample sizes, primary dependent variables, and key statistical tests were chosen in advance and were pre-registered (see OSF: https://osf.io/yweut/). This study was approved by the relevant Institutional Review Board.

We collected data from two separate sources (in-person vs. online) so that we could reach a large sample size in a reasonable amount of time. Here, and for both subsequent experiments, our “online” studies were administered with the supervision of a parent or guardian but were not supervised by an experimenter. We provided parents with explicit instructions about how to administer the task and ensured they understood that they were not meant to intervene. As a protective measure, we pre-registered that we would thoroughly compare the online and in-person samples to check for any statistical differences. There were no statistically detectable differences between our in-person and online samples so we merged the two samples. We report the relevant statistics in the Results section of each experiment.

#### Stimuli.

There were 84 distinct stimuli; 12 exemplars for each of 7 distinct topologies. Half of the exemplars for each topology were made up of 3 line segments and the other half were made up of 4 line segments. We counted line segments in terms of the minimal number of straight lines that would be needed to draw the image ignoring any vertices. For instance, a “T” could be thought of as one line resting on top of another line, or it could be thought of as three lines joining at a central point. For our purposes here, a “T” would count as only two line segments. In terms of [T-junctions-Holes], the 7 unique topologies were: [0–0], [0–1], [1–0], [1–1], [2–0], [2–1], [3–1]. ([0–0] refers to an item with zero T-junctions and zero holes, like an “L”, whereas [1–0] refers to an item with one T-junction and zero holes, like a “T”.) All stimuli are available on our OSF page (and are described using the same notation). Each item was presented inside of a circle, which was designed to be approximately 1 inch in diameter on a typical computer display.

#### Procedure.

Children were introduced to the “odd-one-out” task in which they would see a 3 × 2 grid of six ‘mysterious shapes’ and were asked to identify which one was not like the others. They were given no additional information about how they were meant to identify the odd-one-out. There was no time limit on their responses, and they were not instructed to hurry. Children tested in person made their responses by pointing to their answer, at which point an experimenter would submit the response. Children tested online could make their response by using the mouse to click on their chosen item, or having the parent do so. Children were told that by playing the game they could collect ‘gems’ to exchange for a prize at the end. After each trial, children were shown an image of a gem. Children completed two representative practice trials, the data from which were not recorded, before beginning the task.

To ensure the task duration was suitable for children, a subset of twelve comparisons was selected. The twelve comparisons were: [0–0 vs. 0–1], [0–0 vs. 1–0], [0–1 vs. 1–1], [1–0 vs. 1–1], [1–1 vs. 2–1], [2–0 vs. 1–0], [2–0 vs. 2–1], [2–1 vs. 3–1], [2–0 vs. 0–0], [1–1 vs. 3–1], [2–1 vs. 0–1], [3–1 vs. 0–1]. These 12 were selected to provide a range of comparisons which vary in the number of differences across the two topologies. Each of the twelve comparisons was presented a total of six times: three times with one of the topologies in the majority, and three times with the other topology in the majority (i.e., for the comparison [0–0 vs. 0–1], there were two trials for which 0–0 was the odd-one-out and two trials for which 0–1 was the odd-one-out). Additionally, one-third of trials consisted only of items with three line segments; another one-third of trials consisted of items with only four line segments; and a final one-third of trials consisted of an equal mix of items with three and four line segments. Other than these constraints, the specific exemplars that were chosen for each topology, as well as the locations in which they appeared, were fully randomized. This meant that, for a given comparison [such as 0–0 vs. 0–1], participants might see different exemplars. The trials were divided into three blocks of 24 trials each. The trial types were not blocked in any meaningful way; only to allow children a short break between segments. Children who participated in person, if they were especially restless, were occasionally given a sticker between segments.

For children who participated remotely, detailed instructions were given to the parents/guardians regarding how to administer the study. They were told not to help the child make a choice for any reason, but that they could provide encouragement. Because parents were not aware of the specific goals of the study, and there were no objectively correct answers, it is unlikely that a parent would have intervened on a child’s behalf. We compared the online and in-person samples directly to confirm that there was no parental interference (see Results and Discussion).

### Results and Discussion

**Figure 2.**
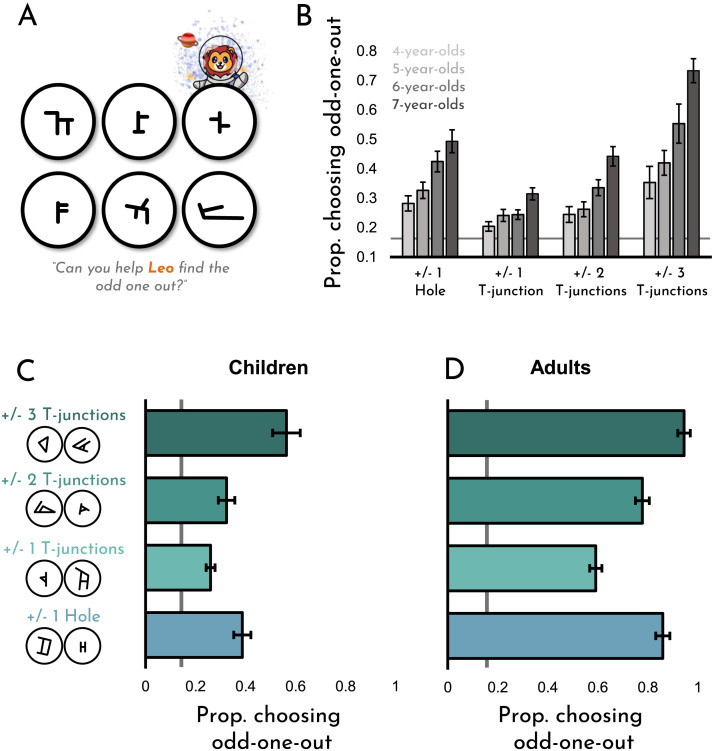
**(A)** A depiction of a typical trial in Experiment 1. The topological odd-one-out in this case is the bottom-right item, which, unlike the other items, has only one T-junction. **(B)** Overall data, broken down by item type and age group. **(C)** A summary of the overall data, which can be compared against **(D)** comparable data from adults, taken from Yousif and Brannon (2024). Error bars represent ±1 *SE*.

Results from this experiment can be seen in Figure 2. As is evident from the figure, children were well above chance (16.67%) in discriminating topological differences. This finding was true for all comparison types (±1 hole: *t*(99) = 12.02, *p* < .001, *d* = 1.20; ±1 T-junction: *t*(99) = 8.52, *p* < .001, *d* = .85; ±2 T-junctions: *t*(99) = 9.72, *p* < .001, *d* = .97; ±3 T-junctions: *t*(99) = 11.83, *p* < .001, *d* = 1.18), as well as for each of the twelve unique comparisons (*p*s < .05). As is evident from the figure, differences in holes were more salient than T-junctions; participants were better at detecting a difference of 1 hole than differences of one T-junction (*t*(99) = 8.72, *p* < .001, *d* = .87) or even two T-junctions (*t*(99) = 5.18, *p* < .001, *d* = .518), but worse than differences of three T-junctions (*t*(99) = 6.13, *p* < .001, *d* = .61). Additionally, as seen in Figure 2B, though performance gradually improved from ages four to seven (*r*(98) = .53, *p* < .001), even the youngest children were above chance for all trial types (*t*s(99) > 2.35, *p*s < .03, *d*s > .47).

Performance for in-person and online participants did not differ (*t*(98) = .072, *p* = .94, *d* = .02). Data broken down by group and by age can be seen in the supplementary materials on our OSF page (see Figure S1A).

While overt topological differences (such as the presence or lack of a hole) may seem simple to distinguish, it is important to note that children were given no instructions whatsoever regarding how they were meant to characterize the items, and could have attempted to differentiate the items based on a number of other features. Furthermore, as can be seen in Figure 2D, many of these comparisons can be challenging even for an adult with formal education. Even so, children were above-chance for even the most difficult items tested.

## EXPERIMENT 2: MATCHING TASK

The previous task revealed that children can make discrimination judgments based on topological structure. We next implemented a task that requires explicit similarity judgments. Children were presented with a sample item and required to choose which of two items was more similar to the sample. On each trial, one of the choice items was a topology-match: It mirrored the topology of the sample item but was altered through minor adjustments such as repositioning or rotating a line segment. The second item was a topology-mismatch: It was topologically different from the sample but was objectively more similar in terms of surface features (see Methods for an in-depth explanation). This setup created a conflict between surface-level similarity and underlying topological equivalence, in that one item visually resembled the sample more closely while the other matched its fundamental topological structure. Our question was whether children, like adults, would match items based on topological structure rather than surface-level similarities.

### Methods

#### Participants.

136 children (*M*_age_ = 5.97; *SD*_age_ = 1.14) completed the task; approximately evenly distributed across each age from four (four years, zero days) to seven (seven years, 364 days). We preregistered that we would collect data from 100 participants total, 25 from each age group. However, towards the conclusion of data collection, we unexpectedly obtained data from a large number of children all at once in a short span of time. Rather than throwing out all of these data, we decided to stray from our pre-registered plan and include more information. Note that our findings in no way depend on these additional data points. The results are robust for all possible subsets of 100 participants. The age breakdown was as follows: 33 four-year-olds, 38 five-year-olds, 32 six-year-olds, and 33 seven-year-olds. 76 of the participants completed the task in person, and the other 60 completed the task online via the Children Helping Science platform (Scott & Schulz, 2017). Per our pre-registered exclusion criteria, an additional 5 participants were excluded due to overt negligence or inattention. This study was approved by the relevant Institutional Review Board.

#### Stimuli.

**Figure 3.**
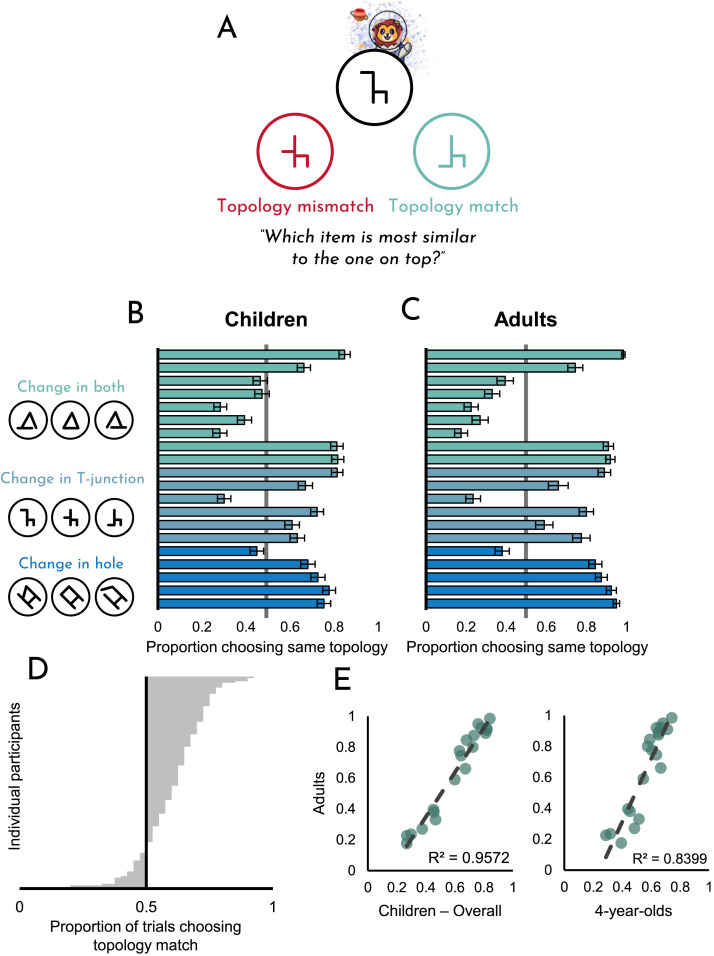
**(A)** A depiction of a typical trial in Experiment 2. **(B)** Overall data, broken down by item compared against **(C)** comparable data from adults, taken from Yousif and Brannon (2024). **(D)** Overall data broken down by participant. **(E)** Correlations between adults’ selections and children’s selections. Error bars represent ±1 *SE*.

Each trial included (1) a sample item, (2) a topology-mismatch (a variation of the sample item with a fixed change, via translation or rotation, that disrupted topology), and (3) a topology-match (a variation of the sample item with twice as much physical change as the topology-mismatch but which maintained the sample topology as the sample). In other words, the topology-mismatch was always more physically similar to the sample (in the sense that the relevant part of the stimulus ‘moved’ less far) than the topology-match was, but the topology-match retained the sample’s topological form. For instance, in Figure 3A, the sample item is shown at the top of the display, and the two variants are shown on either side beneath it. The topology-mismatch was created by moving the leftward horizontal line halfway down the vertical line, resulting in a shape that has a different topology from the sample. The topology-match was created by moving that same leftward horizontal line fully down the vertical line. This topology-match involved that same line moving by twice as much, but the result was an item that shared the topology of the sample. For all twenty unique items that we created, the topology-match always differed from the sample by twice as much as the topology-mismatch in exactly this way. The stimuli were designed by a research assistant (with instructions from the authors) with no knowledge of the hypotheses.

#### Procedure.

On each trial, the sample (one of 20 items) appeared at the top of the screen. Two test items were presented below the sample, on the left and right sides of the screen. Children were prompted to indicate which item was most similar to the item on top. Children tested in person made their responses by pointing to their answer, at which point an experimenter submitted the response. Children tested online could make their response by using the mouse to click on their chosen item, or having the parent do so. Children were told that by playing the game they could collect ‘gems’ to exchange for a prize at the end. After each trial, children were shown an image of a gem. Each of 20 items was shown twice for a total of 40 trials, divided into 2 blocks of 20 trials each. Of those 2 unique presentations, each of the topology-matched items in a set appeared as the sample item (at the top of the display). The different-topology item (i.e., the single item in the set which had a distinct topology from the other items) never appeared as the sample item. The order of these trials was fully randomized within each participant. Participants completed two representative practice trials, the data from which were not recorded, before beginning the task.

### Results and Discussion

Results from this experiment can be seen in Figure 3. As is evident from the figure, children preferentially matched based on topology rather than surface features (*t*(135) = 9.47, *p* < .001, *d* = .81). This was independently true for 13 of the 20 unique item sets (*p*s < .003). Of the seven for which children did not exhibit a topology preference, only four of them were significantly different from chance. Figure 3D shows the overall preference for each participant; of the 136 children tested, 106 of them favored the topology matches overall (binomial test, *p* < .001).

Overall, performance for in-person and online participants did not differ (*t*(98) = .01, *p* = .99, *d* < .01). Data broken down by group, age, and testing location can be seen in the supplementary materials on our OSF page (see Figure S1B).

In separate work (Yousif & Brannon, 2024), we have tested how adults match these exact same items in an almost-identical task. Therefore, we can ask not only whether children prefer topology-matched items over feature-matched items, but also how their preferences compare to adult preferences on an item-by-item basis. For reference, the adult data can be seen in Figure 3C and the correspondence between the adult data and the child data can be seen in Figure 3E. The correlation between choices made by adults and children is remarkably high, *r*(18) = .98, *p* < .001. Even the correlation between adult choices and choices made by the youngest cohort of children (4-year-olds), is still highly robust, *r*(18) = .92, *p* < .001.

Why do we observe such stability in the matching task given that we observed marked change in performance from age 4 to 7 in the odd-one-out task in Experiment 1? We cannot say for sure. However, it seems likely that given the greater difficulty of the task used in Experiment 1 that the change in performance as a function of age reflected domain general skills. In Experiment 1, children were asked to consider six letter-like forms simultaneously and select only one that was not like the others. In contrast, Experiment 2 involved a binary choice, and, at most, processing three items simultaneously. In addition, the stimuli themselves were simpler in Experiment 2: All three items shown on the screen at one time were variants of each other. It seems probable, then, that Experiment 2 demanded fewer domain general cognitive resources (attention, working memory, etc.). Viewed this way, the developmental change occurring between four and seven years old in Experiment 1 may not be specific to children’s understanding of topology.

Overall, both children and adults prefer the topology matches to the feature matches. However, for those items that children *do not* prefer the topology matches, adults *also* do not prefer the topology matches. This means that, whatever primitive shape features are guiding similarity judgments, there is remarkable stability in shape representations across development. These strong correlations raise the provocative possibility that there are ‘core’ topological or geometric features that shape spatial understanding from an early age and are largely unaffected by formal education with geometric concepts. While topological features are likely one kind of ‘core’ feature, there are surely others; more needs to be done to understand what other features guide similarity judgments like these.

For different sorts of stimuli, children are unsure whether to match them by shape or by topology. Kenderla et al. (2023) found that in a noun-extension task, children aged two to seven years old were equally likely to match an object based on its shape rather than its topology. Children were shown a 2D shape that was given a specific name (e.g., a “toma”) and then asked which of three objects shared that name. One of the three objects had the same contours but a different topology (as defined by the presence or absence of a hole); another had different contours but the same topology; and a final distractor object had distinct contours and topology. These findings suggest that children’s understanding of objects is unlikely to solely depend on topological features, raising interesting questions about when and why topology matters relative to traditional Euclidean features like length angle and distance.

Combined with the results of Experiment 1, these results suggest that not only can children discriminate and match items based on topology, but that knowledge of these topological features is deeply ingrained (in the sense that even as knowledge of formal geometric concepts evolves through formal education, discrimination and identification of forms based on topological structure is highly consistent).

## EXPERIMENT 3: TOPOLOGY AND GEOMETRY ODD-ONE-OUT TASK

In a final experiment, we investigated children’s understanding of both topological and geometric concepts through another odd-one-out task. The goal of the experiment was to compare children’s sensitivity to topological relations with their sensitivity to geometric properties (see, e.g., Dehaene et al., 2006; Dillon et al., 2013). We created sets of stimuli that each contained one item that differed from the rest either due to a topological or geometric feature. We asked children to identify which item was not like the others and compared their performance on trials with a topological vs. a geometric deviant. Through this experiment, we aimed to discern whether or to what extent network topology is related to other notions of topology, or other “core” geometric knowledge.

### Methods

#### Participants.

200 children (*M*_age_ = 5.99; *SD*_age_ = 1.18) completed the task, made up of 50 children of each age from four (four years, zero days) to seven (seven years, 364 days). This sample size was chosen to be comparable to our intended sample size of Experiments 1 and 2, except that we wanted to maximize our ability to detect meaningful differences in our factor analysis. We ran some tests on simulated data and decided that around 150 participants would be sufficient for our analysis. To maintain some level of consistency with the prior experiments, and to be conservative, we elected to simply double our previous sample sizes. 100 of the participants completed the task in person, and the other 100 completed the task online via the Children Helping Science platform (Scott & Schulz, 2017). Per our pre-registered exclusion criteria, an additional 5 participants were excluded due to inattention. Additionally, to have a direct point of comparison, we collected data from 50 adult participants via Prolific. The task was identical to what the children completed except that we removed the use of a cartoon character in the instructions and throughout the task. There were no exclusions from the adult sample. This study was approved by the relevant Institutional Review Board.

#### Stimuli.

**Figure 4.**
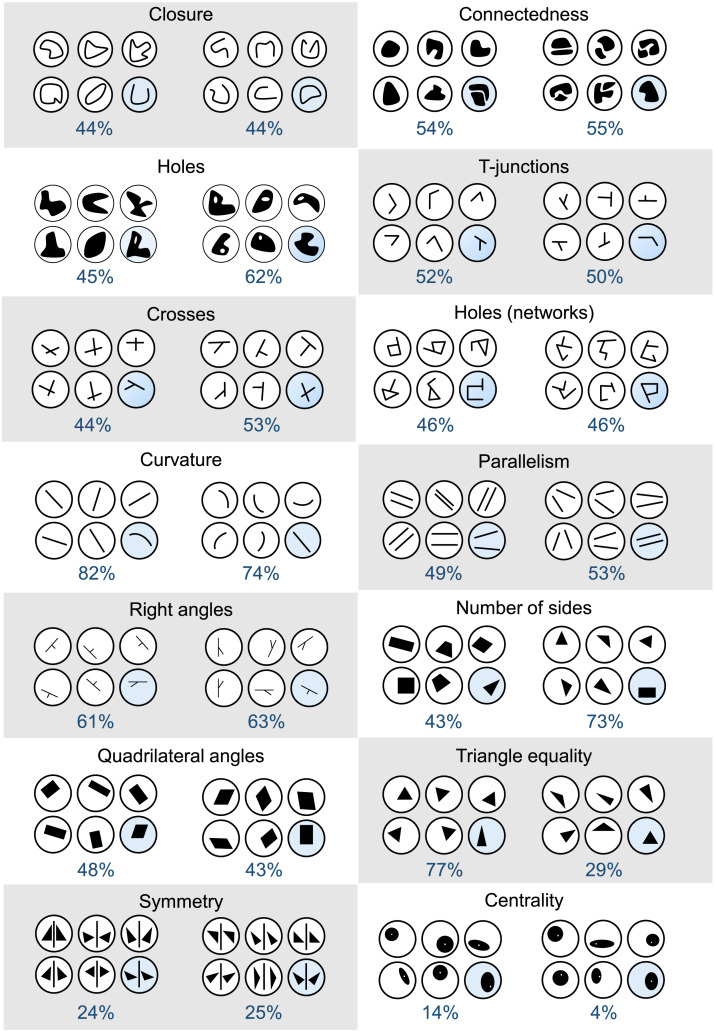
Each item from Experiment 3 and the percentage of times that children chose the odd one out (shown in blue). Here the odd one out is always shown in the bottom right, but during the task the location of the odd one out was randomized.

There were 14 categories of stimuli including six sets examining different topological features (closure, connectedness, holes [in objects], T-junctions, crosses, and holes [in networks]) and eight sets examining different geometric features (curvature, parallelism, symmetry, centrality, triangle equality, the presence of right angles, the number of sides, quadrilateral structure). These features were chosen based on prior work (Dehaene et al., 2006). An example of each type of stimulus can be seen in Figure 4. Each of the six stimuli on each trial was presented inside of a circle, which was designed to be approximately 1 inch in diameter on a typical computer display. Within each trial, all items were made up of the same number of line segments.

#### Procedure.

The procedure was identical to Experiment 1, except as noted. Given the six topological features and eight geometric features examined, there were 14 total trial types. Each trial type had two variations: One in which a particular feature was in the majority, and another in which that same feature was in the minority. For instance, in looking at curvature, one variation showed five curved lines and one straight line, and another variation showed five straight lines and one curved line. Each of these variations was repeated twice, for a total of 56 trials. The trials were divided into two blocks of 28 trials each.

### Results and Discussion

**Figure 5.**
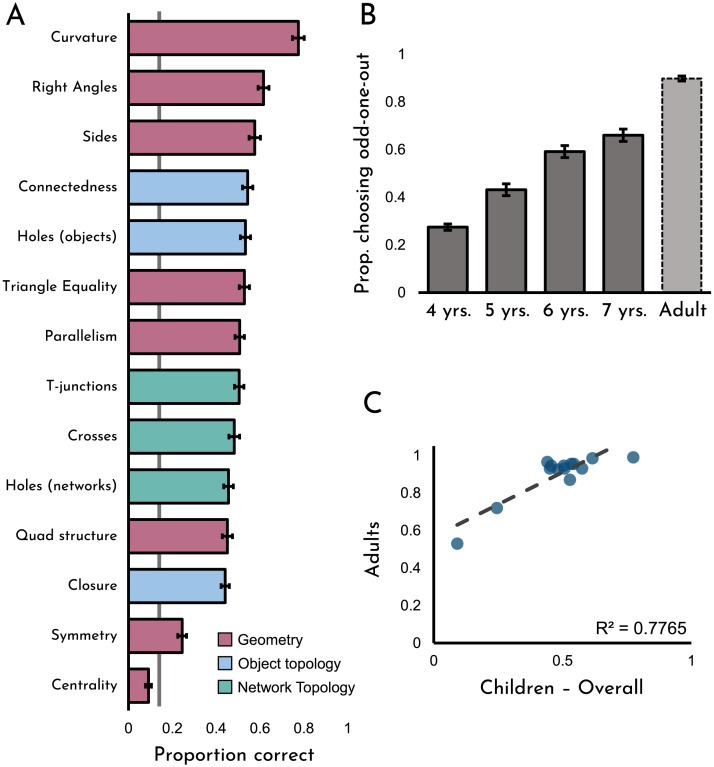
**(A)** Overall data for Experiment 3 separated by item. **(B)** Data separated by age group. **(C)** A correlation between adults’ responses and children’s responses for each of the 14 items. Error bars represent ±1 *SE*.

Results from this experiment can be seen in Figure 5. As is evident from the figure, children were generally above chance in discriminating both topological and geometric differences (*t*(199) = 20.65, *p* < .001, *d* = 1.46). Consistent with prior work (Dehaene et al., 2006; Dillon et al., 2013), even four-year-old children were above-chance (*t*(49) = 8.15, *p* < .001, *d* = 1.15). Figure 5A shows children’s performance collapsed over age for each of the 14 distinct trial types. Children performed above chance for all of the topology concepts, and all but one of the geometry concepts. Performance was highest on the geometric concepts of curvature, triangle equality, and the presence of right angles, and the topological concepts of connectedness and holes (in objects). Children performed more poorly for the symmetry and centrality comparisons. However, we hasten to add that overall performance is likely to be influenced partially by idiosyncrasies of our stimuli; for instance, in retrospect we realized that the “centrality” items were not as visually discriminable as we would have liked.

Overall, performance for in-person and online participants did not differ (*t*(198) = .86, *p* = .39, *d* = .12). Data broken down by group, age, and testing location can be seen in the supplementary materials on our OSF page (see Figure S1C).

Figure 5B shows performance over all trial types at each age, including the performance of adults on the same stimulus set (the full data broken down by stimulus type and age can be seen in Figure S2 on our OSF page). As can be seen in the figure, performance gradually improved from ages four to seven, with the performance of the seven-year-olds most closely resembling that of the adults. Figure 5C shows the correspondence between the adult data and the overall child data on this task. As is evident from the figure, adults and children make these shape discriminations in a consistent manner (*r*(12) = .88, *p* < .001). Additionally, even the performance of the four-year-old children displayed consistency with the adult performance (*r*(12) = .54, *p* = .046), suggesting that knowledge of both geometric and topological features is deep-rooted and stable from a young age.

Per our preregistered plan, we ran an exploratory factor analysis on the children’s data for the 14 different items tested. The aim of this analysis was to test whether, for instance, all of the topological items (including those in the original stimulus set) might cluster together, separately from all the geometric items. However, it failed to reveal any interpretable patterns of that sort. The factor analysis revealed one factor that explained 43% of the variance (with factor loadings from .58 to .78). The addition of a second factor explained only a further 4% of the variance. The first factor was comprised of 12 of the 14 properties tested—all but symmetry and centrality, which also happened to be the two items which exhibited the worst overall performance (for both children and adults). This finding could be interpreted as evidence that the propensity to differentiate all item types stems from a single, general capacity for representing many different primitive spatial forms. However, we think this analysis should be interpreted with caution, insofar as there are no other factors to compare against. We have reported it here only because it was part of our preregistered analysis plan.

## GENERAL DISCUSSION

From a young age, children exhibit an intuitive grasp of topological structure. Children as young as four demonstrated an ability to distinguish topological differences among various shapes, performing well above chance across all comparison types (Experiment 1). This ability improved considerably with age, with older children (ages 6–7) displaying near-adult-like proficiency. Children also preferred to match forms based on topology rather than based on surface features, with their choices being almost perfectly correlated with those of adults (Experiment 2). Indeed, their ability to identify topological forms seems comparable to their ability to identify basic geometric properties like centrality, perpendicularity, and parallelism (Experiment 3). Furthermore, the remarkable similarity in the responses of children and adults (Experiments 2 and 3) strongly suggests that there are basic building blocks of spatial representation that are early developing and stable throughout the lifespan.

### Stability Across Development

Perhaps the most intriguing finding here is not that children can distinguish items based on their topology or even that children preferentially match items based on topology rather than surface features. Instead, the most striking result here may be that children match items in almost exactly the same way that adults do (Experiment 2). This was evident in the high correlation between the aggregated performance of children and adults (*r* = .98) which held even when we only considered the youngest children tested (4-year-olds; *r* = .92).

Neither children nor adults always matched on topology. In practice, this means that topology alone is not sufficient to explain how people evaluate form similarity. Yet the high correspondence between adults’ and children’s choices suggests that, regardless of what features guide a given choice, there is remarkable stability in spatial understanding from early childhood onward.

This developmental stability presents an opportunity for future work. It could be valuable to understand what features are guiding similarity judgments in those cases where adults and children both match based on something other than topological similarity. Perhaps understanding what factors are guiding children’s choices will help to reveal additional building blocks of spatial representation (see Yousif, 2022; Yousif & Keil, 2021). What’s clear from the present results is that topological features like T-junctions and holes are likely to be one key component of these basic spatial concepts.

### Core Knowledge of Topology Versus Geometry

In the last twenty years or so, there has been interest in understanding the basic building blocks of spatial representation (i.e., “core” geometric properties; see, e.g., Dehaene et al., 2006). Much emphasis has been placed on Euclidean features like length, angle, and distance as the basis for human geometric knowledge (see, e.g., Hermer & Spelke, 1994; Lee et al., 2012; Yousif & Lourenco, 2017). However, here we’ve shown that topological features also feature prominently in how both children and adults evaluate spatial relations. What defines the topological relations studied here is their unique insensitivity to Euclidean properties like distance, length, and angle. It seems unlikely, then, that Euclidean geometry is the sole basis of human spatial representation—a conclusion that is bolstered by a wide range of work in developmental psychology (see, e.g., Huey et al., 2023; Kenderla et al., 2023), perception (Yousif & Brannon, 2025), and spatial navigation (Byrne, 1979; Chrastil & Warren, 2014; Kim & Doeller, 2024; Manley, 2016; Moar & Bower, 1983; Warren et al., 2017).

Interestingly, topology seems to cross-cut multiple domains of core knowledge, including objects (Chen, 1982; Kibbe & Leslie, 2016), forms (Yousif & Brannon, 2024), and even number (Franconeri et al., 2009; He et al., 2015; Yousif & Brannon, 2025). In other words, while it seems that topology represents a functional, important kind of knowledge—one that influences everything from how we perceive network forms to how we enumerate sets—it is not clear where such knowledge fits into the “core knowledge” picture (see Spelke, 2022; Spelke & Kinzler, 2007). Future theoretical perspectives may wish to clarify where topology fits in the broader picture of human knowledge^1^.

Perhaps one reason to believe that topological knowledge is related to other forms of geometric knowledge is that we failed to find a clear difference between the two in Experiment 3. Though we believe these results should be interpreted with caution (see the discussion of Experiment 3), an exploratory factor analysis revealed that a single component was primarily responsible for performance on both geometric and topological trials (Experiment 3). This pattern implies that children’s use of geometric properties may be closely related to their use of topological properties—perhaps part of the same core system of spatial representation. This is a hypothesis worth exploring further in future work.

That said, the topological concepts studied here may be a tool for more than mere spatial representation. Other forms of information can be represented using basic topological features like holes and T-junctions. For instance, one can imagine a social group in which Allie is friends with Beth who is friends with Carol and Dave; in this example, Beth is a “T-junction” between Allie and Carol and Dave. It is not hard to imagine how representing relations in this way could be useful, even in the absence of any spatial information. In this way, network topology could be thought of as a *language of relations*—a way of efficiently representing how things relate to one another, abstracted away from any spatial detail (see Yousif & Brannon, 2024). This is to say that topological relations may be an important building block of human thought, even beyond the domain of spatial representation.

### Shape Skeletons

Drawing inspiration from work on “shape skeletons” (Ayzenberg et al., 2019; Feldman & Singh, 2006), Spelke (2022) argues that form analysis and object classification depend on hierarchically branching representations. Our stimuli bear obvious resemblance to shape skeletons. Given that even infants are sensitive to the skeletal structure of objects (Ayzenberg & Lourenco, 2022), one may wonder whether children’s sensitivity to topological network structures just reflect their known sensitivity to skeletal descriptions of space. We think the answer is “not exactly”.

First, it is worth noting that our stimuli do not have much hierarchical structure. They are designed to emphasize the junctions at which branches occur; there are not many cases in our stimulus set where there are branches from other branches. Instead, our work focuses on what happens at the point where a branch does occur: We show that children are sensitive to different kinds of branches that may occur on an object. Second, shape skeletons like medial axes are a computational description of an object’s shape, whereas network topology offers a way of describing the resulting skeletal forms. The key insight from recent work on skeletal descriptions of shape is that the visual system can (and spontaneously does) extract such skeletal structures. How those representations are compared and disambiguated requires a topological perspective. What branches of a skeletal representation matter? Which axes can be trimmed or pruned? What skeletal features are most diagnostic of an object’s unique shape? These are all topological questions. In this way, one might view the present work as an exploration of how children understand the skeletal structures that underlie object representation.

### Conclusion

Our findings reveal not only that children are broadly sensitive to topological relations, but that their similarity judgments of forms which vary in their topology strongly resemble the judgments of adults. Although much emphasis in prior work has been placed on sensitivity to various aspects of Euclidean geometry, here we show that children are also sensitive to certain aspects of topological representation. These findings shed light on the basic building blocks of spatial representation and highlight the need for understanding many different kinds of representational forms.

## ACKNOWLEDGMENTS

For help with data collection, we thank Shalom Chen, Laura Grosh, Hannah Joo, Naomi Kim, David Koestler, and Sophie Shao. For critical feedback, we thank Sam Clarke, Veronique Izard, Melissa Kibbe, Chuyan Qu, and all the members of the Developming Minds Lab at the University of Pennsylvania.

## DATA AVAILABILITY STATEMENT

Pre-registrations, stimuli, raw data and other supplementary materials are available on our OSF page: https://osf.io/yweut/.

## Note

^1^ One might question whether the effects documented here truly constitute a form of “knowledge” as opposed to merely perceptual sensitivity. We believe this raises a broader question: Might “core knowledge of geometry” (Dehaene et al., 2006) reflect perceptual content rather than knowledge *per se*?
